# PERSONALITY FUNCTIONING FEATURES OF CLINICAL ANXIETY AND COVID-19 ANXIETY AMONG OLDER ADULTS

**DOI:** 10.1093/geroni/igac059.2373

**Published:** 2022-12-20

**Authors:** Lisa Stone, Colleen Mock, Daniel Segal

**Affiliations:** University of Colorado Colorado Springs, Colorado Springs, Colorado, United States; University of Colorado Colorado Springs, Colorado Springs, Colorado, United States; University of Colorado Colorado Springs, Colorado Springs, Colorado, United States

## Abstract

**Introduction:**

Anxiety is a significant mental health problem among older adults and is commonly comorbid with personality disorders (PD). However, specific relationships between personality functioning (a proposed feature of PDs) and late-life anxiety remain unclear. This study examined relationships between two models of personality functioning with late life clinical anxiety and COVID-19 anxiety. Method: Older adults (n = 222) completed the Geriatric Anxiety Scale (GAS), Coronavirus Anxiety Scale (CAS), Levels of Personality Functioning Scale-Self-Report (LPFS-SR), and Severity Indices of Personality Problems-Short Form (SIPP-SF).

**Results:**

The GAS and CAS were significantly correlated to all LPFS-SR and SIPP-SF domains with large effect sizes (> .78); higher clinical and COVID-19 anxiety was associated with increased personality dysfunction. In regressions, the LPFS-SR domains significantly accounted for 65% of variance in the GAS and 59% of variance in the CAS. Identity and Self-Direction were the strongest predictors of each anxiety scale, with Empathy also significantly related to coronavirus anxiety. The SIPP-SF domains significantly accounted for 65% of variance in the GAS and 58% of variance in the CAS. Responsibility and Social Concordance were the strongest predictors of each anxiety scale, with Self-Control also significantly related to clinical anxiety. Discussion: Results indicate theoretically-supported and meaningful overlap between clinical and COVID-19 anxiety with personality dysfunction according to two different models. This extensive overlap questions the extent to which personality functioning differentiates from affective distress. The two personality dysfunction models also differed somewhat in their relationships to anxiety, suggesting the need for further research especially among older adults.

